# Adenine base editing of the *DUX4* polyadenylation signal for targeted genetic therapy in facioscapulohumeral muscular dystrophy

**DOI:** 10.1016/j.omtn.2021.05.020

**Published:** 2021-06-01

**Authors:** Darina Šikrová, Vlad A. Cadar, Yavuz Ariyurek, Jeroen F.J. Laros, Judit Balog, Silvère M. van der Maarel

**Affiliations:** 1 Department of Human Genetics, Leiden University Medical Center, 2333 ZC Leiden, the Netherlands; 2 Leiden University, 2300 RA Leiden, the Netherlands; 3 Leiden Genome Technology Center, Leiden University Medical Center, 2333 ZC Leiden, the Netherlands; 4 Department of Clinical Genetics, Leiden University Medical Center, 2333 ZC Leiden, the Netherlands; 5 National Institute for Public Health and the Environment (RIVM), 3721 MA Bilthoven, the Netherlands

**Keywords:** facioscapulohumeral muscular dystrophy, DUX4, polyadenylation signal, CRISPR-Cas9, base editing, gene therapy

## Abstract

Facioscapulohumeral muscular dystrophy (FSHD) is caused by chromatin relaxation of the D4Z4 repeat resulting in misexpression of the D4Z4-encoded *DUX4* gene in skeletal muscle. One of the key genetic requirements for the stable production of full-length *DUX4* mRNA in skeletal muscle is a functional polyadenylation signal (ATTAAA) in exon three of *DUX4* that is used in somatic cells. Base editors hold great promise to treat DNA lesions underlying genetic diseases through their ability to carry out specific and rapid nucleotide mutagenesis even in postmitotic cells such as skeletal muscle. In this study, we present a simple and straightforward strategy for mutagenesis of the somatic *DUX4* polyadenylation signal by adenine base editing in immortalized myoblasts derived from independent FSHD-affected individuals. We show that mutating this critical *cis*-regulatory element results in downregulation of *DUX4* mRNA and its direct transcriptional target genes. Our findings identify the somatic *DUX4* polyadenylation signal as a therapeutic target and represent the first step toward clinical application of the CRISPR-Cas9 base editing platform for FSHD gene therapy.

## Graphical abstract

**Figure:**
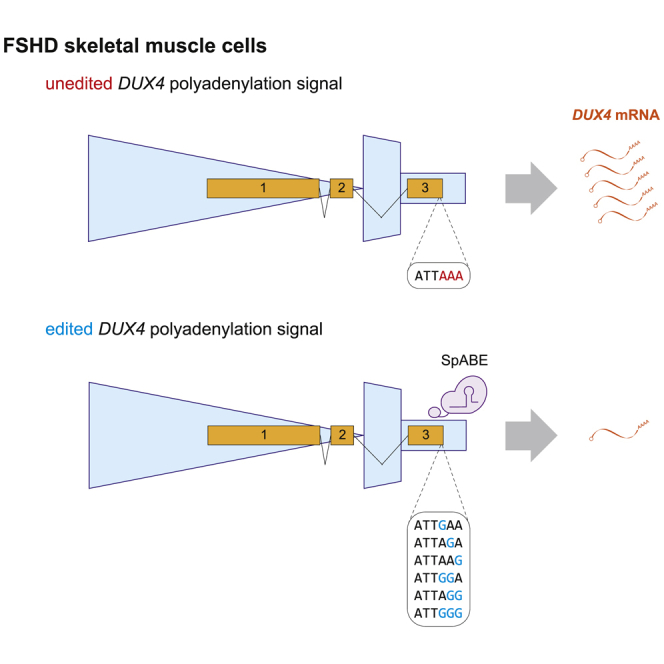

## Teaser

To test the therapeutic potential of adenine base editing in facioscapulohumeral dystrophy, we targeted the *DUX4* polyadenylation signal and achieved efficient *DUX4* mRNA downregulation in FSHD muscle cell cultures.

## Introduction

Facioscapulohumeral muscular dystrophy (FSHD is a hereditary skeletal muscle disorder that typically becomes manifest around the second decade of life and progresses with high inter- and intra-familial variability.^1^, ^2^, ^3^ It is believed that this variability in disease progression and severity can be partially explained by the underlying epigenetic mechanism of the disease, being a failure to establish and/or maintain a repressive chromatin structure of the D4Z4 macrosatellite repeat at 4q35 in somatic cells. This leads to a variegated expression of the D4Z4 repeat-encoded *DUX4* gene in muscle cells.^4^ DUX4 is a pioneer transcription factor that under physiological conditions is expressed in keratinocytes,^5^ testes,^4^ and thymus^6^ and in cleavage stage embryos, where it drives zygotic genome activation.^4^^,^^7^, ^8^, ^9^ When misexpressed in muscle cells, it disrupts, among others, the bona fide muscle transcriptome.^10^^,^^11^

The repressive chromatin environment of the D4Z4 locus in somatic cells is likely established by a repeat-mediated epigenetic silencing mechanism that partly depends on the D4Z4 repeat unit copy number.^12^ There are two genetically distinct but overlapping forms of FSHD: FSHD type 1 (FSHD1; OMIM: MIM158900) and FSHD type 2 (FSHD2; OMIM: MIM158901).^13^^,^^14^ The more common form, FSHD1, is caused by a shortening of the D4Z4 repeat to a size of 1–10 units,^15^ whereas in FSHD2 the repeat size is within the lower range of healthy individuals (9–20 D4Z4 units). In the latter case, *DUX4* de-repression is caused by a malfunction of D4Z4 chromatin modifiers.^16^, ^17^, ^18^ Most FSHD2 individuals can be explained by heterozygous mutations in the gene encoding for the Structural Maintenance of Chromosomes flexible Hinge Domain-Containing protein 1 (SMCHD1),^17^ a protein involved in, among other pathways, epigenetic inactivation of the X chromosome in mammals.^19^, ^20^, ^21^, ^22^, ^23^ A small number of *SMCHD1* mutation-negative FSHD2 families have been reported in which mutations in the genes encoding for the chromatin modifiers DNA Methyltransferase 3B (DNMT3B) or Ligand Dependent Nuclear Receptor Interacting Factor 1 (LRIF1) were shown to cause D4Z4 chromatin relaxation and *DUX4* expression in skeletal muscle.^16^^,^^18^

In addition to D4Z4 chromatin relaxation, the genetic background of the 4q subtelomere is critically important for FSHD manifestation. There are two equally common variants of this subtelomere, termed 4qA and 4qB;^24^ however, only the 4qA variant is associated with the disease.^25^^,^^26^ This is due to a sequence difference immediately distal to the distal D4Z4 unit, where the 4qA allele contains an additional 260 bp sequence termed pLAM that creates the third exon of *DUX4* with a functional ATTAAA polyadenylation signal (PAS) in somatic cells. Such genetic prerequisite for developing FSHD is supported by the finding that a contraction of the highly homologous D4Z4 repeat on chromosome 10 (10q26) does not lead to FSHD despite the presence of the pLAM sequence. However, this sequence contains a single-nucleotide polymorphism (SNP) in the corresponding *DUX4* PAS sequence (ATTAAA → ATCAAA), which renders it non-functional.^27^ The critical importance of this *DUX4* PAS sequence was recently corroborated with the identification of two chromosome 10q-linked FSHD families in which the distal end of the disease-associated contracted D4Z4 repeat on chromosome 10, including the pLAM sequence, originated from chromosome 4.^28^ Likewise, 4qB chromosomes lack the pLAM sequence altogether, and, consequently, a D4Z4 repeat contraction on this genetic background does not lead to the development of FSHD.^26^

Previously, it has been shown by different approaches, including the application of antisense oligonucleotides, DNA nucleases and U7 small nuclear RNA (snRNA), that interference with the usage of the endogenous 4qA *DUX4* PAS in myogenic cells derived from FSHD patients results in transcriptional downregulation of *DUX4* and its target genes,^29^, ^30^, ^31^, ^32^, ^33^ further emphasizing the necessity of the annotated 4qA *DUX4* PAS for proper 3′ end processing of *DUX4* pre-mRNA and suggesting that interfering with its usage is sufficient to alleviate the FSHD expression signature in myogenic cells.

Currently, there is no cure for FSHD and because of the underlying genetic character of the disease, CRISPR/Cas9 genome editing could be a promising tool for its treatment . Unfortunately, because of the repetitive nature of the *DUX4* gene (every D4Z4 unit contains one copy of the *DUX4* open reading frame [ORF]), a straightforward Cas9 nuclease-mediated knockout strategy might lead to multiple breaks, trigger genomic instability, and result in cell death as has been shown for targeting multicopy genomic regions.^34^ Therefore, a different approach is required. The novel RNA-programmable base editing system, which consists of a wild-type (WT) tRNA adenosine deaminase (TadA) and an artificially evolved version of TadA (TadA∗) fused as a dimer to the D10A nicking version of *Streptococcus pyogenes* Cas9 (nSpCas9), hereafter referred to as nSpABE, enables robust adenine to guanine substitution without reliance on homology-directed repair (HDR) or introduction of double-stranded DNA breaks.^35^ Such editing system has already been shown to faithfully edit the desired nucleotides also in postmitotic cells such as neurons^36^ or skeletal muscle cells.^37^^,^^38^ In this study, we aimed to take advantage of this system to demonstrate that the 4qA *DUX4* PAS can be efficiently disrupted with this approach, resulting in downregulation of *DUX4* transcript levels in FSHD myogenic cells.

## Results

### Validation of sgRNA targeting *DUX4* polyadenylation signal in HAP1 cells

**Figure 1:**
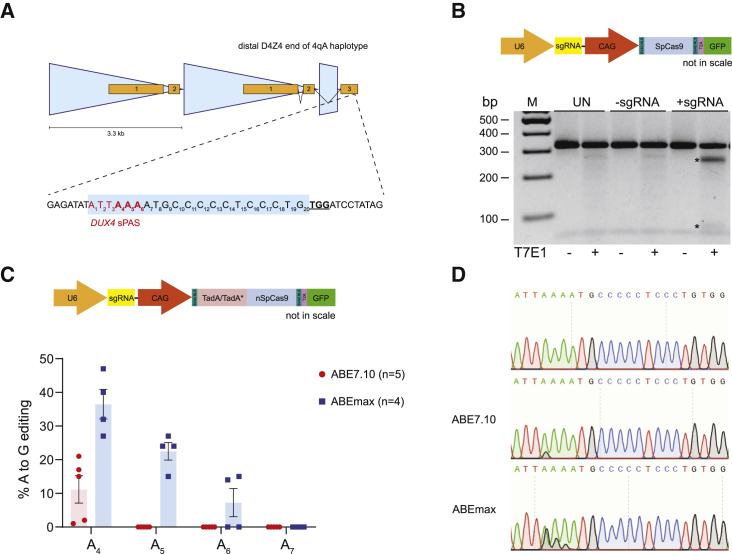
Adenine base editors can edit the *DUX4* PAS (A) Schematic representation of the distal end of the 4qA-derived D4Z4 macrosatellite repeat (each blue triangle represents one D4Z4 repeat unit) including the adjacent downstream sequence containing the polyadenylation signal of *DUX4* in exon 3 (*DUX4* exons are indicated by orange boxes) and zoom in on the sequence to be targeted by the adenine base editor. The sgRNA protospacer is outlined in the blue box, the PAM site for SpCas9 is underlined in bold and the *DUX4* PAS sequence (ATTAAA) is in red font with adenines that can be targeted by the adenine base editor in bold. (B) Schematic map of the pX458 vector for simultaneous sgRNA and SpCas9 nuclease expression (top). Result of the T7E1 assay performed on HAP1 cells that were transfected with the pX458 vector expressing the sgRNA targeting the *DUX4* PAS together with a plasmid encoding for puromycin resistance to select for transfected HAP1 cells (bottom). Untransfected cells (UN) or cells transfected with no sgRNA-containing vector (−sgRNA) served as negative control. Asterisks mark the T7E1 cleavage products. (C) Schematic map of the modified all-in-one pX458 vector encoding for the adenine base editors (top). Editing efficiency was assessed in HAP1 cells for the ABE7.10 and ABEmax versions of the adenine base editor. The A→G editing efficiency was calculated from Sanger sequencing tracks with EditR^39^ for each adenine in the editing window. Graph shows mean ± SEM of at least four independent biological replicates (dots). (D) Representative Sanger sequencing tracks for ABE7.10- or ABEmax-mediated editing of the *DUX4* PAS used for quantification.

In myonuclei, the FSHD disease gene *DUX4* is transcribed from the distal unit of the D4Z4 repeat on the 4qA subtelomere, where its transcripts are stabilized by a PAS in exon 3. The adjacent SpCas9 protospacer adjacent motif (PAM) site (TGG) downstream of this PAS allows for the design of an single guide RNA (sgRNA) that places the last three adenines of the *DUX4* PAS (ATTAAA) in the activity window of nSpABE (Figure 1A). To test whether this sgRNA can effectively direct the Cas9 machinery to the locus of interest, we first performed a T7E1 assay on HAP1 cells transfected with the sgRNA and a human codon-optimized SpCas9 nuclease. Despite having a repeat of 25 D4Z4 units on chromosome 4, which is most probably compacted into a dense chromatin structure perhaps hindering the interaction of the DNA with CRISPR/Cas9, we could clearly detect cleavage of the intended locus (Figure 1B). To evaluate A→G base editing of the *DUX4* PAS, we used a one-vector system for delivery of all adenine base editing components. HAP1 cells were individually transfected with two variants of the all-in-one vector in which the CAG promoter drives expression of the SpCas9 nickase fused to either the ABE7.10 or the ABEmax version of the adenine base editor, hereafter referred to as nSpABE7.10 and nSpABEmax, respectively (Figure 1C) and examined for A→G edits at the *DUX4* PAS site by Sanger sequencing. In nSpABE7.10-transfected cells, we could detect on average 11.2% ± 3.6% of A→G conversion for the adenine at position 4 of the protospacer (A_4_) as assessed by Sanger sequencing. We did not detect editing of adenines at positions 5 to 7 (A_5-7_) despite these adenines still fitting into the reported activity window of nSpABE7.10.^35^ In nSpABEmax-transfected cells, we achieved more efficient adenine base editing at A_4_ (36.5% ± 3.8%) as well as at downstream adenines A_5_ (22.5% ± 2.25%) and A_6_ (7.3% ± 3.6%), which is in agreement with a previous report that nSpABEmax is superior to nSpABE7.10 in terms of editing efficiency and processivity.^40^

Next, we assessed adenine editing of the *DUX4* PAS, using the ABEmax in combination with two other Cas9 orthologs, SaCas9 and CjCas9, since their cognate PAM sites, NNGRRT and NNNVRYM, respectively, are in the vicinity of the *DUX4* PAS such that adenines on the forward or reverse strand in the *DUX4* PAS could be amenable to adenine base editing (Figure S1A). We used the same all-in-one vector architecture as was used for nSpABEmax, including the same linker length, and the new constructs are hereafter referred to as nSaABEmax and nCjABEmax (Figure S1B). Surprisingly, both constructs failed to exert adenine base editing activity at the *DUX4* PAS in HAP1 cells based on evaluation by Sanger sequencing as was done for SpABE7.10 and SpABEmax (data not shown).

### Base editing of *DUX4* PAS in patient-derived immortalized FSHD1 and FSHD2 myoblasts

**Figure 2:**
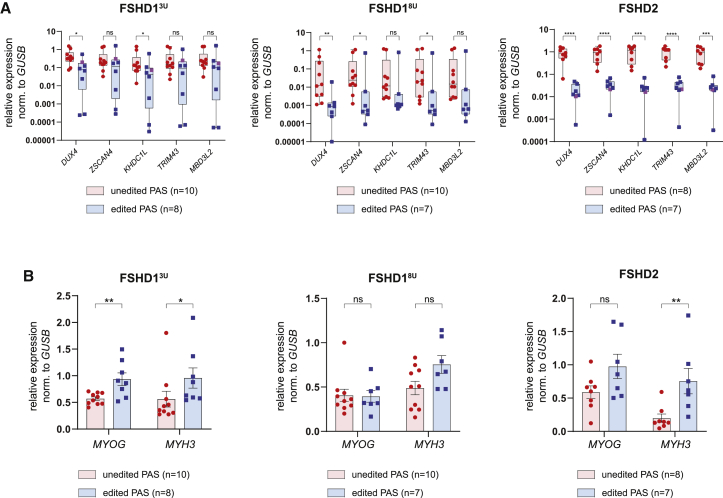
Adenine base editing of the *DUX4* PAS reduces expression of *DUX4* and its target genes in FSHD myogenic cells derived from polyclonal cultures (A) mRNA levels as assessed by qRT-PCR of *DUX4* and four DUX4 target genes (*MBD3L2*, *ZSCAN4*, *TRIM43*, and *KHDC1L*) in PAS unedited versus edited clones derived from two FSHD1 and one FSHD2 cell lines differentiated into myotubes. Statistical significance was calculated with unpaired two-tailed t test (ns, non-significant; ∗p < 0.05, ∗∗p < 0.01, ∗∗∗p < 0.001, ∗∗∗∗p < 0.0001) on log2 transformed expression values to correct for skewed distribution. Expression values normalized to *GUSB* as housekeeping gene are plotted. Line represents mean, and whiskers represent min and max value. Individual dots represent individual clones; the two violet clones carry a deletion affecting the *DUX4* PAS. (B) mRNA levels of two myogenic markers (*MYOG* and *MYH3*) for all unedited and edited clones of all three FSHD cell lines are plotted. Statistical significance was calculated with unpaired two-tailed t test (ns, non-significant; ∗p < 0.05, ∗∗p < 0.01, ∗∗∗p < 0.001, ∗∗∗∗p < 0.0001). Bars represent mean ± SEM, with individual clone expression values plotted as individual dots.

To explore the effect of the mutated PAS on *DUX4* steady-state transcript levels, we carried out base editing in FSHD patient-derived immortalized myoblasts, since HAP1 cells do not express *DUX4*. We used three different FSHD myogenic cell lines with different genetic characteristics, D4Z4 methylation status and *DUX4* expression levels (Figures S2A and S2B). We selected one FSHD2 cell line that has a heterozygous missense mutation in SMCHD1 (K204E) combined with an 11-unit-long 4qA D4Z4 repeat and two FSHD1 cell lines, one with a 3-unit-long 4qA D4Z4 repeat (FSHD1^3U^) and one with an 8-unit-long 4qA repeat (FSHD1^8U^). Shorter D4Z4 repeats are generally correlating with lower D4Z4 methylation levels,^41^ a more severe FSHD phenotype and a worse prognosis,^2^ whereas repeats in the upper size limit of FSHD1 typically show a higher incidence of familial non-penetrance and a milder disease presentation.^3^^,^^42^ Furthermore, we chose cell lines heterozygous for 4qA and 4qB to facilitate unequivocal assignment of successful editing of the FSHD allele, except for FSHD1^8U^, which carries two variant alleles of 4qA (with the healthy allele being of the 4qA161L variant and the FSHD allele of the 4qA161S variant).^43^ However, these two allelic variants of 4qA161 can be distinguished by the presence of a SNP (Figure S3A). Clonal cell cultures from all three cell lines were genotyped for the *DUX4* PAS after transfection with nSpABEmax and single-cell sorting of GFP^+^ cells. Untransfected cells underwent the same sorting procedure to obtain clones with a WT PAS sequence to ensure the same experimental conditions and population doublings between compared groups. Successfully edited clones showed a plethora of A→G editing outcomes (Figure S3A). We also obtained one clone from the FSHD1^3U^ and one clone from the FSHD2 cell line in which the editing attempt resulted in small deletions fully or partially encompassing the *DUX4* PAS (Figure S3A). *DUX4* steady-state mRNA levels were measured as well as those of four well-established DUX4 target genes (*ZSCAN4*, *KHDC1L*, *TRIM43*, and *MBD3L2*)^11^^,^^44^ serving as an indirect readout for DUX4 transcription factor activity. The steady-state mRNA levels of *DUX4* and its target genes were reduced in all three cell lines upon editing of the *DUX4* PAS under proliferating (Figure S3B) as well as differentiating (Figure 2A) conditions. Since it has been shown that *DUX4* expression increases during myogenic differentiation,^45^ we analyzed the expression of early (*MYOG*) as well as late (*MYH3*) myogenic markers by qRT-PCR to rule out the possibility that lower *DUX4* levels were due to reduced differentiation potential of edited clones (Figure 2B). On the contrary, edited clones showed equal if not slightly increased myogenic differentiation, which is in agreement with previous findings that DUX4 inhibits myogenic differentiation, thereby lowering its levels would improve differentiation.^10^ However, unedited clones showed a high variability in *DUX4* expression levels and those of its target genes ranging from 1 order of magnitude in the FSHD1^3U^ and FSHD2 lines up to 3 orders of magnitude in clones derived from the FSHD1^8U^ line. Such high expression variability thus makes it difficult to confidently determine the effect of *DUX4* downregulation conferred by base editing.

### Reducing the clonal variability in *DUX4* expression

Since D4Z4 displays highly variable transcriptional activity between individuals^46^ and across cells from the same individual (this study), a behavior that is also described for genomic loci known as metastable epialleles^47^ of which their epigenetic profile is stochastically established in early embryogenesis, we hypothesized that starting the editing from a monoclonal cell culture rather than a polyclonal culture may resolve a large part of inter-clonal variability in *DUX4* expression. This would facilitate a better comparison of *DUX4* levels between *DUX4* PAS pre-editing and post-editing clones in the absence of large expression variability at WT baseline. We therefore first tested the “mitotic stability” of *DUX4* expression by deriving new daughter clones from two clones showing different levels of *DUX4* expression (referred to as DUX4^high^ and DUX4^low^) originating from the FSHD1^8U^ line, as it showed the highest *DUX4* expression variability. Indeed, after resorting, new single-cell derived cultures exhibited more homogeneous *DUX4* and DUX4 target gene (*ZSCAN4* and *MBD3L2*) expression levels comparable to the parental clone as measured by qRT-PCR (Figure S4).

**Figure 3:**
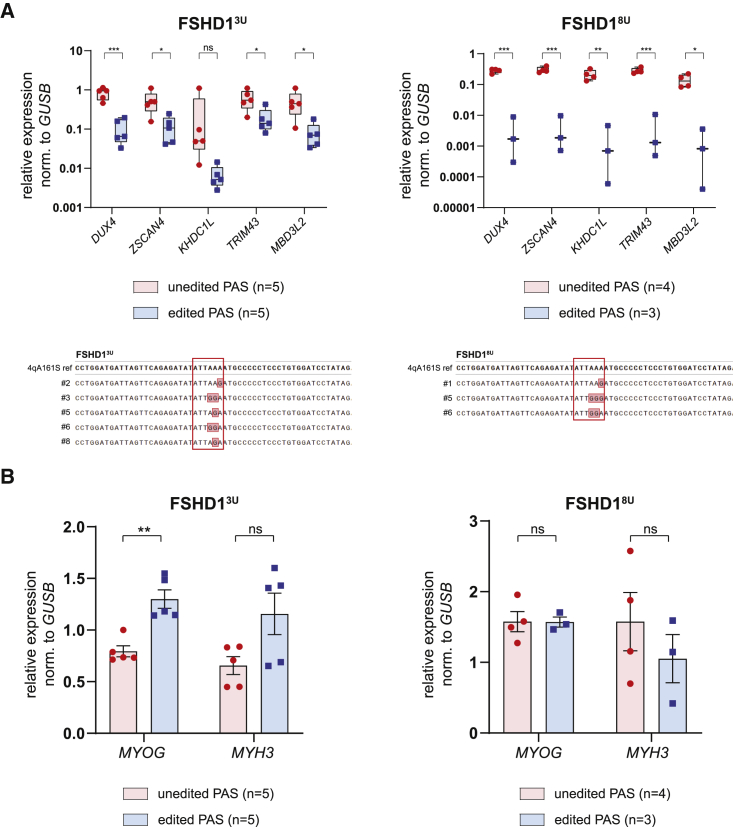
Adenine base editing of the *DUX4* PAS reduces expression of *DUX4* and its target genes in FSHD myogenic cells derived from monoclonal cultures (A) mRNA levels as assessed by qRT-PCR of *DUX4* and four DUX4 target genes (*MBD3L2*, *ZSCAN4*, *TRIM43*, and *KHDC1L*) in *DUX4* PAS unedited versus edited subclones derived from two clones with different FSHD1 cell line origins (top). Genotypes of edited clones aligned to the reference WT sequence with the *DUX4* PAS are highlighted in a red rectangle, and red colored bases denote mismatches (bottom). (B) mRNA levels of two myogenic markers (*MYOG* and *MYH3*) for unedited and edited clones from (A). Statistical significance was calculated with unpaired two-tailed t test (ns, non-significant; ∗p < 0.05, ∗∗p < 0.01, ∗∗∗p < 0.001, ∗∗∗∗p < 0.0001). Bars represent mean ± SEM, with individual clone expression values plotted as individual dots.

We selected one unedited DUX4^high^ clone derived from either the FSHD1^3U^ or the FSHD1^8U^ cell line and repeated the editing procedure to obtain new *DUX4* PAS unedited and edited clones. As expected, deriving new unedited clones from a monoclonal culture resulted in lower *DUX4* expression variability between clones, with clones carrying an edited *DUX4* PAS showing significantly reduced *DUX4* steady-state mRNA levels as well as DUX4 target gene levels (Figure 3A). Again, the reduced *DUX4* expression levels could not be attributed to a difference in myogenic differentiation, as shown by comparable expression of the two myogenic differentiation markers between edited and unedited clones (Figure 3B). Interestingly, editing the *DUX4* PAS seems to have a more negative impact on *DUX4* mRNA levels in FSHD1^8U^ (∼1,000-fold downregulation) than in cells from FSHD1^3U^ line (∼10-fold downregulation).

### Editing of the *DUX4* PAS induces alternative pre-mRNA cleavage and polyadenylation

**Figure 4:**
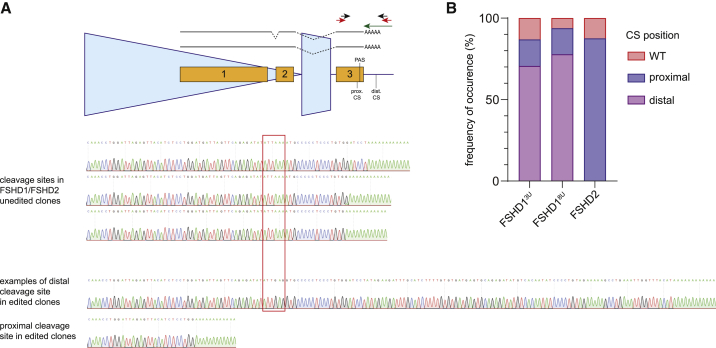
Editing of the *DUX4* PAS induces alternative pre-mRNA cleavage site (A) Schematic of the terminal D4Z4 repeat unit with short ending (4A161S haplotype) showing the design of 3′ RACE experiment to determine the cleavage and polyadenylation site of *DUX4* mRNA in the edited clones. Two known *DUX4* mRNA isoforms are depicted with splicing or retention of intron 1 (top). Arrows represent primers used for oligo-dT reverse transcription (green), first PCR (red), and second nested PCR (black). The identified proximal and distal cleavage sites, for which Sanger sequencing traces are provided, are marked. Sanger sequencing tracks (bottom) show representative examples of 3′ ends of *DUX4* mRNA in *DUX4* PAS unedited and edited FSHD1/FSHD2 clones. The red rectangle outlines the *DUX4* PAS sequence. Three different CSs were identified in unedited clones (as reported previously^31^), while different shifts in CSs were identified in edited clones. One representative Sanger sequencing track is shown for each CS choice. (B) Barplots representing the frequency of occurrence of different CSs identified in *DUX4* PAS edited clones with respect to WT CSs from ≥4 clones for each condition.

Previously, it was shown that hindering the *DUX4* PAS with phosphorodiamidate morpholino oligomers (PMOs) causes a redirection of the *DUX4* pre-mRNA cleavage site (CS) ∼40 nt upstream of its canonical CS despite the absence of a recognizable alternative PAS motif in the upstream sequence.^31^ Since base editing of the *DUX4* PAS does not completely abolish *DUX4* expression, we tested if the mutated PAS is still being used for *DUX4* transcript termination, albeit less efficiently, or if alternative PASs/CSs are being used. Using a semiquantitative 3′ rapid amplification of cDNA ends (3' RACE) to identify 3′ UTR sequences of *DUX4* mRNAs from unedited and edited clones derived from all three FSHD immortalized cell lines from Figure 2A, we detected three different CSs 16–24 nt downstream of *DUX4* PAS in close proximity to each other in unedited cells (Figure 4A), as was previously described.^31^ In edited clones, however, two different shifts in the CS occur, either proximally or distally to the canonical CS (Figure 4A & B, Table S3). Interestingly, the FSHD2 edited clones strictly used the proximal CS, the same one as reported by Marsollier et al.^31^ after using PMOs against the *DUX4* PAS region, whereas the distal CS switch is predominant in the FSHD1 clones independent of their 4qA permissive allele size (Figure 4B). Moreover, opposite to the single proximal CS being used after PAS editing, the distal CS is not as deterministic, since we observed multiple different 3′ ends in FSHD1 edited clones. Of note, the small proportion of *DUX4* mRNAs using the canonical CS position in FSHD2 clones is coming from the single clone that carries a partial deletion of *DUX4* PAS. Despite the clear shift in the CS upon *DUX4* PAS editing, we could not detect a nearby PAS-like sequence (±100 nt from original PAS) which could explain the CS shifts. Overall these data show that *DUX4* PAS base editing prevents proper 3′ end formation of the *DUX4* transcript.

### Off-target analysis by targeted next generation sequencing

**Figure 5:**
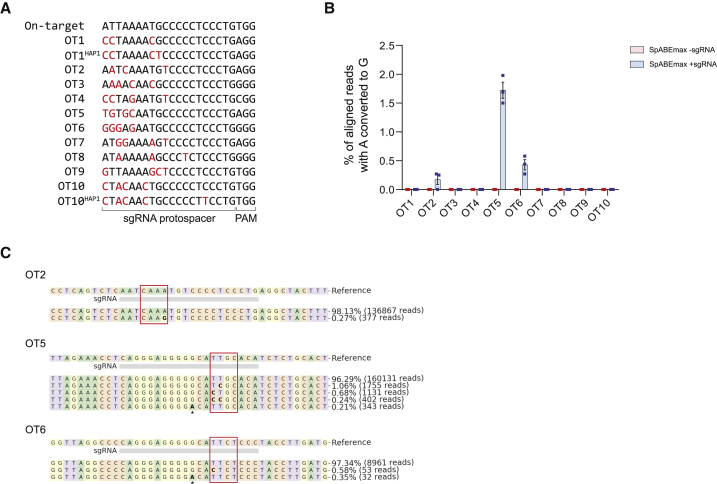
sgRNA-dependent off-target analysis in HAP1 cells (A) DNA sequences of 10 predicted off-target sites identified by CRISPOR.^48^ Nucleotide mismatches compared to the *DUX4* PAS target sequence are highlighted with red font. Two off-target sites (OT1 and OT10) carried an extra mismatch in HAP1 cells as compared to the reference sequence obtained from GRCh38. (B) Editing frequencies at predicted off-target sites were assessed in HAP1 cells that were transfected with nSpABEmax and either with or without *DUX4* PAS targeting sgRNA. The A→G editing efficiency was assessed by amplicon next generation sequencing and analyzed with CRISPResso2.^49^ Graph shows mean ± SEM of 3 independent biological replicates. (C) Representative allele frequencies of three off-target sites (OT2, OT5, and OT6) with the highest editing outcome are shown. OT5 and OT6 sequences are shown in forward orientation, while sgRNA targets the reverse complement strand. The editing windows are highlighted in the red box. Only allele frequencies of at least 0.1% were considered. The mutation rate in the G homopolymer (marked by asterisk) preceding the editing window was not included in the editing frequency calculation plotted in (B) since it occurred also in the control samples and was more likely introduced either during PCR steps or Illumina sequencing itself rather than in an sgRNA-dependent fashion.

To explore potential off-target effects, we used the CRISPOR prediction tool^48^ to identify genomic sites that have a sequence homology to the sgRNA used for targeting the *DUX4* PAS. This resulted in the identification of 227 potential off-target (OT) sites, of which none are predicted to target polyadenylation signals of other genes. Only 3 are predicted to target coding sequences, however, with low off-target scores due to the number and position of individual mismatches (Table S4). We further filtered predicted off-target sites by the following criteria:(1) having up to 4 mismatches outside of the PAM region and the seed region of the sgRNA, (2) containing at least one adenine in the editing window of nSpABEmax, and (3) representing a single copy locus. Based on these criteria, we performed targeted next generation sequencing on 10 selected potential off-target sites in DNA samples obtained from HAP1 cells that were transfected with nSpABEmax with or without sgRNA targeting the *DUX4* PAS from Figures 1C and 1D (Figure 5A). At 7 out of 10 examined sites, deep sequencing did not reveal any appreciable increase in A→G transitions within or near the editing window as compared to the control samples (Figure 5B). However, the nucleotide sequences of OT1 and OT10 contained a SNP in the HAP1 genome, producing an extra mismatch in the sgRNA protospacer (Figure 5A). Therefore, their off-target potential might be higher in genomes that do not contain this mismatch. At three sites, OT2 (chr6: 13,331,126–13,331,148), OT5 (chr12: 2,444,719–2,444,741), and OT6 (chr2: 218,831,310–218,831,332), we detected editing efficiencies of 0.17%, 1.72%, and 0.43% of adenines within the editing window, respectively (Figures 5B and 5C). None of the three affected OT sites resides in coding regions. OT2 is in an intergenic region ∼2 kb upstream of the *TBC1D7* gene, while OT5 and OT6 map to intron 3 of *CACNA1C* and intron 2 of *PRKAG3*, respectively. Both genes, *CACNA1C* and *PRKAG3*, are expressed in skeletal muscle according to the Human Protein Atlas,^50^ but neither edit is predicted to affect the splicing of these genes when modeled with the Alamut software. In summary, these results show that sgRNA-dependent off-target DNA editing is likely rare.

## Discussion

So far, therapeutic attempts for FSHD have been mainly focused on oligonucleotide- or small molecule-based transient modulation of *DUX4* levels.^51^^,^^52^ Three recent studies focused on gene therapy approaches that inhibit the production of full-length *DUX4* mRNA.^32^^,^^33^^,^^53^ Two of these studies used CRISPR/Cas9 strategies, either employing a standard Cas9 nuclease to introduce deletions affecting the *DUX4* PAS by HDR with a provided template^32^ or using Cas9 coupled to a transcriptional inhibitor domain to repress *DUX4* expression^53^. The third study used custom U7 nuclear RNAs (snRNAs) to mask important regulatory features of *DUX4* mRNA maturation such as splice sites and the *DUX4* PAS.^33^ In this study, we demonstrate the use of a CRISPR/Cas9-based genome editing application to directly modify the *DUX4* locus while avoiding DNA double-strand breaks. We show that by using an adenine base editor we can target and disable one of the important genetic prerequisites for FSHD manifestation, the *DUX4* somatic polyadenylation signal. We were able to successfully edit the *DUX4* PAS with SpCas9-based base editors nSpABE7.10 and nSpABEmax, with the latter showing higher editing efficiency, which is in agreement with previous reports.^54^ Fusing ABEmax to two other Cas9 orthologs, namely SaCas9 and CjCas9, has previously been shown to also result in adenine editing activity.^54^, ^55^, ^56^ However, we did not observe adenine to guanine conversion at the *DUX4* PAS when using such fusion proteins in HAP1 cells as determined by Sanger sequencing. The T7E1 assay nevertheless did show evidence for recruitment of the SaCas9 nuclease to the *DUX4* PAS site (Figure S1C), suggesting that the complex can be recruited to the *DUX4* PAS but that the nSaABEmax fusion protein is likely not efficient at this site. Previously, a lower editing efficiency has been reported for nSaABEmax as compared to nSpABEmax,^54^ which could explain our findings. Recently, a new version of the adenine base editor, termed ABE8e, was described.^55^ When paired with a variety of Cas effectors, including SaCas9, it demonstrated a further enhanced editing efficiency. Therefore, coupling ABE8e to SaCas9 might result in successful adenine base editing of the *DUX4* PAS. In addition, such a fusion construct would be more favorable compared to the SpCas9 construct because of its smaller size, which could facilitate the use of the adeno-associated viral (AAV) system for its *in vivo* delivery and testing. Alternatively, an AAV split system could be used for *in vivo* delivery of SpABEmax or SpABE8e. Indeed, such an approach has been already tested for delivering base editors to a range of tissues,^37^^,^^57^ reaching 20% editing efficiency in skeletal muscle tissue.^57^ Since published strategies were aiming at whole body delivery and were not optimized for skeletal muscle targeting or expression, further optimization by using a tissue-specific promoter and a muscle-trophic AAV serotype might increase the editing efficiencies in the skeletal muscle. On the other hand, the failure to detect editing of the *DUX4* PAS with nCjABEmax might be attributed to a suboptimal nearby PAM sequence (5′-AATCATC-3′) that was predicted for the targeting. We identified this PAM site based on the PAM consensus sequence (5′-NNNVRYM-3′) reported by Yamada et al.^58^ Another study by Kim et al.^59^ reported a slightly different PAM consensus sequence (5′-NNNNRYAC-3′) for CjCas9 targeting that is more refined and differs from the sequence that we used for deriving our sgRNA. Moreover, such a fusion construct has not been characterized in depth yet; therefore there is no knowledge about its precise editing window or its efficiency.

As anticipated, editing of the *DUX4* PAS in immortalized myogenic lines obtained from different FSHD-affected individuals resulted in lower *DUX4* mRNA levels and lower DUX4 transcription factor activity as indirectly measured by the steady-state mRNA levels of its target genes. We could not determine if editing more adenines at once or if editing an adenine at a particular position in the *DUX4* PAS motif results in a more profound *DUX4* downregulation, since multiple clones with the same editing outcome would be required to confidently assess this. Nevertheless, we show that even a single adenine substitution is sufficient to negatively impact proper 3′ end processing of the *DUX4* transcript. To our surprise, mutating the *DUX4* PAS in this manner does not completely abolish the production of polyadenylated *DUX4* transcripts as opposed to the situation on chromosome 10, which might suggest the presence of other *cis* modifiers acting as regulators of *DUX4* expression than just the previously recognized SNP in 4q/10q *DUX4* PAS motif. These *cis* factors are likely in linkage disequilibrium with the *DUX4* PAS considering the exclusive linkage of FSHD with the presence of a *DUX4* PAS. Interestingly, in two independent FSHD1 clonal cell lines we observed different steady-state *DUX4* mRNA level reduction upon editing (Figure 3A). Since we cannot correlate this outcome to the initial *DUX4* expression levels, to the nucleotide edit at the *DUX4* PAS, or to the methylation levels at the targeted region, this outcome may be a reflection of its suspected role as metastable epiallele, as the chromatin environment has also been suggested to influence PAS usage efficiency.^60^^,^^61^ Such individualistic response will require further studies to elucidate its mechanism and to be able to predict the benefit of this approach for FSHD patients.

In addition, the study by Joubert et al. reported the use of either paired transcription activator-like effector nucleases (TALENs) or paired CRISPR/Cas9 nucleases to excise the *DUX4* PAS sequence with the aim of incorporating a mir-1 sequence by HDR in immortalized myoblasts.^32^ This approach yielded only 2 successfully edited clones out of 227 (0.8%). In contrast, with our approach we achieved 30/163 successfully edited immortalized myoblast clones (nearly 20%) across five different experiments including three different FSHD cell lines (Table S5). Nevertheless, despite the limited number of successfully edited clones in the Joubert study, they also observed reduced, but not abolished, *DUX4* and DUX4 target gene levels and a switch in the *DUX4* mRNA cleavage and polyadenylation site, which corroborates our findings. The increased editing efficiency in our study could be explained by the fact that adenine base editors act independently of the HDR pathway, a pathway that is only available in S and G2 phases of the cell cycle. This cell cycle-independent feature of the ABE system makes it a viable candidate for its future *in vivo* translatability. The main bottleneck for adenine editing efficiency may therefore very well be the optimal delivery of editing components to skeletal muscle tissue.

One of the main concerns for the use of genome editing platforms is their potential off-target effect. Adenine base editors have been shown to suffer from sgRNA-dependent off-target DNA editing, albeit to a lesser extent than cytidine base editors.^62^ In this study, we detected at least three sites that were edited in an sgRNA-dependent fashion but to a much lesser extent than the intended site. We observed ∼23-fold more efficient editing at the A_4_ position of the on-target site, i.e., 40% as assessed by Sanger sequencing in gDNA samples that were used also for the inspection of off-target editing in HAP1 cells, as compared to the most efficiently edited off-target site (OT5, 1.7%) as assessed by Illumina short read sequencing. Additionally, off-target editing of cellular RNAs by adenine base editors has been reported.^63^ However, we have not explored this particular side effect of nSpABEmax. In any case, both DNA and RNA off-target activity of adenine base editors can be minimized by making use of further engineered adenine deaminases^55^^,^^64^^,^^65^ linked to higher-fidelity Cas9 versions^66^, ^67^, ^68^ and modified sgRNAs^69^ and by reducing exposure time and/or effector molecule concentrations by employing different delivery strategies such as in the form of ribonucleoprotein particles.^55^^,^^70^ The specificity of the adenine base editing approach for *DUX4* PAS targeting should therefore be carefully evaluated to ensure safety in case of its therapeutic application.

Base editors have been already used to achieve efficient gene silencing by targeting *cis*-regulatory elements important for proper gene expression by either introducing in-frame stop codons,^71^^,^^72^ mutating a start codon,^73^ or disrupting splice sites.^74^^,^^75^ Since deviations from the canonical PAS hexamers generally reduce their cleavage and polyadenylation efficiency,^76^ we explored how many polyadenylation signals genome-wide would be amenable for such an editing approach. We focused on the two most widely used hexameric motifs, namely AATAAA and ATTAAA, as they constitute ∼80% of all identified polyadenylation signals (Figure S5A). These PAS motifs can be disrupted with adenine base editors either by modifying any of the adenines of the last three nucleotide positions of the PAS motif on the coding strand or alternatively by targeting the adenine on the non-coding strand that pairs with the middle thymine on the coding strand, leading to its substitution with a cytidine (Figure S5B). Based on these criteria, we established that ∼25% of all PASs with either AATAAA or ATTAAA motifs are editable with nSpABEmax (Figure S5C). However, it should be pointed out that weakening the core PAS motif might not always lead to the expected transcriptional downregulation, since other *cis* auxiliary elements are known to influence the efficiency of PAS usage.^77^ Moreover, alternative polyadenylation is widespread for genes that contain multiple functional PASs;^78^ therefore invalidating only one of them might not be sufficient to achieve an overall desired level of silencing. Rather, since alternative polyadenylation is tissue specific and globally regulated, PAS editing might represent a more refined tool for gene editing in some conditions. Therefore, the utility of this approach requires locus-specific validation. Nevertheless, due to challenging gene structure, *DUX4* represents an excellent candidate for adenine base editing-mediated mutagenesis of its PAS as a means for its expression interference.

## Materials and methods

### Cloning

To create the all-in-one base editing vector pX458-ABE7.10, overlapping PCR products of the TadA dimer from pCMV-ABE7.10 (Addgene #102919), nCas9-SV40 NLS from pX335 (Addgene #42335), and T2A-GFP from pX458 were cloned in pX458 using the AgeI and EcoRI restriction sites. The pX458-ABEmax vector was created by cutting out the TadA dimer together with the N-terminal domain of Cas9 from pX458-ABE7.10 using the AgeI and ApaI sites and replacing it with the PCR-amplified TadA dimer missing the N-terminal domain of Cas9 from the pCMV-ABEmax-GFP vector (Addgene #112101). The pX601-SaABEmax vector was cloned by first creating a new insert consisting of the TadA dimer linked to the N-terminal domain of SaCas9. This was achieved by overlapping PCR amplifications on pCMV-ABEmax (for the TadA dimer) and pX601 (for the SaCas9 domain) during which a D10A mutation was introduced into SaCas9. The resulting PCR product was cloned in pX601 using the XbaI and HindIII sites. The pX601-CjABEmax was created by first mutating the KpnI site upstream of the CAG promoter in the pX601-SaABEmax vector by replacing it with the same PCR fragment containing a KpnI mutation and cloned using XbaI and AgeI. Next, the SaABEmax-T2A-GFP-bGH insert was replaced by CjABEmax-T2A-GFP-bGH, which was produced by overlapping PCRs on pX601-SaABEmax for TadA dimer, pX404 (Addgene #68338) for CjCas9 (D8A mutation was introduced during this PCR step), and pX601-SaABEmax for T2A-GFP-bGH PAS. The final insert was cloned into pX601-SaABEmax via the AgeI and KpnI sites. Further, the SaCas9 sgRNA expression cassette was replaced with an CjCas9 sgRNA expression cassette. The CjCas9 sgRNA expression cassette was assembled by overlapping PCRs on pX601 to amplify the U6 promoter sequence and on the pU6-Cj-sgRNA plasmid (Addgene #89753) to amplify the sgRNA scaffold. The resulting insert was cloned into the pX601-CjABEmax plasmid created in the previous step via the KpnI and NotI sites. All sgRNAs were cloned into their target vector according to the Zhang lab’s protocol.^79^ For the pX458 vector (Addgene #48138) and its adenine base editor derivatives (SpABE7.10 and SpABEmax), the BbsI sites were used, and for the pX601 vector’s derivatives (SaABEmax and CjABEmax) the BsaI sites were used. For optimal transcription from the U6 promoter, an extra G nucleotide was added to the 5′ end of the sgRNA in case the sequence did not start with one already. All constructs were verified by Sanger sequencing. All primers used are listed in Table S1. The following restriction enzymes were used for cloning: AgeI-HF (New England Biolabs, ##R3552), EcoRI (Thermo Fisher Scientific, #ER0271), ApaI (New England Biolabs, #R0114), HindIII (New England Biolabs, #R0104), KpnI-HF (New England Biolabs, #R3142), NotI-HF (New England Biolabs, #R3189), BbsI (New England Biolabs, #R3539), and BsaI (Thermo Fisher Scientific, #ER0291).

### Cell culture and transfection

Detailed information about cell lines used in this study can be found in Table S2. The HAP1 cell line was maintained in IMDM-GlutaMAX (Thermo Fisher Scientific, #31980) supplemented with 10% fetal bovine serum (FBS) (Gibco, #10270106) and 1% (v/v) penicillin-streptomycin (Gibco, #15140). Immortalized myoblast cell lines 073iMB (FSHD1^8U^) and 200iMB (FSHD2) were a kind gift from Prof. S. Tapscott, Fred Hutchinson Cancer Research Center. The 2402iMB line (FSHD1^3U^) was obtained by immortalizing primary myoblasts, which were a kind gift of Prof. R. Tawil from the University of Rochester, by stable integration of hTERT and CDK4 retroviruses as described previously.^80^ All initial primary myoblast lines originated from the Fields Center for FSHD and Neuromuscular Research at the University of Rochester Medical Center and were obtained following the informed consent after the study had been approved by the relevant institutional review board. All myogenic lines were maintained in Ham’s F-10 Nutrient Mix (Gibco, #31550) supplemented with 20% (v/v) FBS, 1% (v/v) penicillin-streptomycin, 10 ng/mL fibroblast growth factor (FGF)-b (PromoKine, #C-60240), and 1 μM dexamethasone (Sigma-Aldrich, #D2915). Myogenic differentiation was achieved by switching myoblasts at 100% confluency to DMEM (Gibco, #31966021) supplemented with 2% (v/v) KnockOut serum replacement (Gibco, #10828028). All cell lines were maintained at 37°C and 5% CO_2_ and were tested for *Mycoplasma* contamination with the MycoAlert Mycoplasma detection kit (Lonza, #LT07-318) according to the vendor’s instructions. One day prior to transfection, 2 × 10^5^ HAP1 cells were seeded in a 12-well plate. Transfection was performed with 1.5 μg of the base editing vector and 0.5 μg of a vector containing puromycin resistance cassette (AA19_pLKO.1-puro.U6.sgRNA.BveI-stuffer plasmid, a kind gift from Prof. M.A.F.V. Gonçalves, Leiden University Medical Center) using Lipofectamine 3000 (Thermo Fisher Scientific, #L3000008) according to the manufacturer’s instructions. The next day, the medium was replaced with medium containing 0.5 μg/mL of puromycin, and cells were selected for 48 h, after which the medium was replaced again with non-puromycin medium and cells were grown for an additional 72 h, after which they were harvested for subsequent analysis. For myoblast experiments, 3 × 10^5^ myoblasts were seeded in a 6-well plate, and the following day cells were transfected with 2 μg of plasmid DNA using Lipofectamine 3000 (Thermo Fisher Scientific, #L3000008) according to the manufacturer’s instructions. Medium was changed the next day, and cells were harvested for further analysis 72 h after transfection.

### T7E1 cleavage assay

CRISPR/Cas9-induced indels at the targeted locus were examined with the T7E1 cleavage assay. Three days after transfection, cells were harvested in lysis buffer for genomic DNA (100 mM Tris-HCl pH 8.0, 50 mM EDTA pH 8.0, 2% [w/v] SDS) and DNA was extracted by protein precipitation by addition of saturated salt to the solution and subsequent isopropanol precipitation. The target locus was amplified by PCR using DreamTaq (Thermo Fisher Scientific, #EP0701) with the following cycling conditions: 95°C for 5 min followed by 35 cycles of 95°C for 25 s, 67°C for 25 s, and 72°C for 20 s, with a final extension step at 72°C for 5 min. Resulting PCR products were subjected to reannealing in a thermal cycler with the following conditions: 95°C for 5 min followed by cooling down from 95°C to 85°C at 2°C/s and from 85°C to 25°C at 0.1°C/s. After reannealing, 10 μL of PCR product was incubated with T7E1 enzyme (New England Biolabs, #E3321) according to the manufacturer’s instructions. Resulting products were resolved on a 2% Tris-borate-EDTA (TBE) agarose gel with ethidium bromide.

### Fluorescence-activated cell sorting

Cells were trypsinized, collected in their respective culturing media, and spun down, and the cell pellet was resuspended in fluorescence-activated cell sorting (FACS) buffer (10% [v/v] FBS in PBS). Cells were sorted with a BD FACS Aria III cell sorter according to GFP fluorescence, and collected cells were used for further analysis or expansion.

### *DUX4* PAS genotyping and quantification of base editing efficiency

Exon 3 of *DUX4* containing the PAS was amplified from genomic DNA by PCR as described in the T7E1 cleavage assay. The product’s purity was first assessed by an electrophoretic separation on a 2% TBE agarose gel and then extracted from the gel with the NucleoSpin Gel and PCR Clean-up kit (Bioké, #740609) and submitted for Sanger sequencing with the forward primer used in the PCR. Base editing efficiency in the initial test in HAP1 cells was assessed by Sanger sequencing and estimated with Edit-R^39^ (online tool available at http://baseeditr.com/).

### RNA isolation, cDNA synthesis, and qRT-PCR

Cells were harvested in QIAzol lysis reagent (QIAGEN, #79306), and RNA was isolated with the RNeasy mini kit (QIAGEN, #74101) with DNase I treatment according to the manufacturer’s protocol. Oligo-dT-primed cDNA was synthesized from 2 μg of input RNA with the Minus First Strand cDNA synthesis kit (Thermo Fisher Scientific, #K1621). Gene expression was measured with the CFX384 system (Bio-Rad) in technical triplicates using iQ SYBR Green Supermix (Bio-Rad, #1708887). qRT-PCR primers are listed in Table S1. *GUSB* was used as a housekeeping gene.

### 3′ RACE

The 3′ RACE was carried out as reported previously^31^ with minor modifications. The cDNA synthesis was carried out with the Minus First Strand cDNA synthesis kit with modified oligo-dT primer: 5′-GCTGTCAACGATACGCTACGTAACGGCATGACAGTGTTTTTTTTTTTTTTTTTTTTTTTT-3′. The first PCR was performed using 2 μL of cDNA as template in a final volume of 20 μL with AccuPrime *Taq* high-fidelity DNA polymerase (Thermo Fisher Scientific, #1236086) with previously published forward and reverse primers and according to established PCR cycling conditions.^31^ Nested PCR was performed using 2 μL of primary PCR product with AccuPrime *Taq* high-fidelity DNA polymerase with previously published forward and reverse primers and according to established PCR cycling conditions.^31^ Final PCR products were purified from 2% TBE agarose gel and subcloned into the TOPO-TA vector (Thermo Fisher Scientific, #450641). At least 6–8 individual bacterial colonies were screened to determine the *DUX4* mRNA 3′ ends.

### Methylation analysis of *DUX4* exon 3 (FasPAS region) by bisulfite PCR followed by TOPO-TA subcloning

500 ng of genomic DNA was converted with the EZ DNA Methylation-Lightning kit (Zymo Research, #D5030) according to the manufacturer’s protocol. The FasPAS region was amplified from converted DNA with previously published primers (Table S1) using high-fidelity AccuPrime *Taq* DNA polymerase (Thermo Fisher Scientific, #12346086) with the following PCR program: 95°C for 4 min followed by 35 cycles of 95°C for 4 min, 58°C for 20 s, and 72°C for 40 s, followed by a final extension step at 72°C for 5 min. PCR products were purified by electrophoresis and isolated from gel with the NucleoSpin Gel & PCR Clean-up kit (Bioké, #740609) followed by subcloning into the TOPO-TA vector. Plasmid DNA from individual bacterial colonies was sent for Sanger sequencing using the M13R primer, and methylation levels were assessed with BiQ Analyzer software. Methylation lollipop plots were produced with the online QUMA tool (http://quma.cdb.riken.jp/top/index.html).

### sgRNA-dependent off-target analysis using targeted next generation sequencing

Potential off-target sites were predicted by CRISPOR (http://crispor.tefor.net/crispor.py).^48^ Ten predicted off-target sites were chosen based on the MIT specificity score and uniqueness of the region for specific amplification. Genomic regions of interest were amplified with specific primers containing appropriate Illumina forward and reverse adaptor sequences (Table S1). For the first PCR, 100 ng of genomic DNA was used as starting material in a 25 μL reaction further containing 0.4 μM of forward and reverse primer and 12.5 μL of 2× KAPA HiFi HotStart ReadyMix (Kapa Biosystems, #KK2601). PCR reactions were carried out as follows: 95°C for 3 min followed by 27 cycles of 98°C for 20 s, 64°C for 15 s, and 72°C for 15 s, with a final extension step at 72°C for 3 min. This first PCR product was purified with AMPure beads (Beckman Coulter, #A63881) with a 0.8 PCR-to-beads ratio according to the manufacturer’s instructions, and DNA was eluted in 10 μL of EB buffer. A subsequent barcoding PCR was performed in a total volume of 25 μL using 3 μL of purified first PCR product, 2 μL of Illumina barcoding primer mix, and 12.5 μL of 2× KAPA HiFi HotStart ReadyMix. The barcoding PCR was carried out as follows: 95°C for 3 min followed by 7 cycles of 98°C for 20 s, 60°C for 20 s, and 72°C for 20 s, with a final extension step at 72°C for 3 min. PCR products were purified with AMPure beads in a 0.8 PCR-to-beads ratio according to manufacturer’s instructions, and DNA was eluted to 10 μL of EB buffer. The concentration of the final purified amplicons was measured with Qubit, and all amplicons were pooled in equimolar ratio and sequenced on an Illumina MiSeq instrument. Paired-end reads were evaluated for mutations by alignment to the provided predicted off-target sequence using CRISPResso2^49^ (CRISPRessoBatch–batch_settings ‘my_tab_separated_batchfile’–amplicon_seq ‘my_reference_sequence’–base_edit -g ‘my_sgrna_sequence’ -wc −10 -w 20). The effect of intronic mutations on gene splicing was predicted with Alamut Visual software (Interactive Biosoftware, Rouen, France, version 2.15).

### Genome-wide detection of editable polyadenylation signals

In order to find all editable polyadenylation signals in the genome with an AATAAA or ATTAAA motif, we constructed a regular expression that combines the polyadenylation signal motif sequence with a PAM site for SpCas9 (5′-NGG-3′) at appropriate distance from the targeted base so that it falls into the reported activity window of nSpABEmax.^54^ This regular expression was used to find all matching patterns in the human reference genome GRCh38 (https://hgdownload.cse.ucsc.edu/goldenPath/hg38/bigZips/). A similar approach was used to find all occurrences on the reverse complement strand. The results of this search were intersected with a list of known polyadenylation signals (ftp://ftp.ebi.ac.uk/pub/databases/gencode/Gencode_human/release_35/gencode.v35.polyAs.gff3.gz) to obtain the final list of editable polyadenylation signals. We used the ‘famotif2bed’ subcommand of the Fastools (https://fastools.readthedocs.io/en/latest/) package (version 1.0.2) for finding patterns in a reference sequence using regular expressions. All genome arithmetic was done using bedtools (https://bedtools.readthedocs.io/en/latest/) (version 2.27.1). The full procedure is available online (https://github.com/jfjlaros/motif-edit) under the MIT Open Source license.

### Statistical methods

GraphPad Prism software v.8.4.2 was used for calculation of statistics. Sample sizes were not pre-determined prior to experiments, and a concrete statistical test is stated in the respective figure legend.

### Data availability

The sequencing data generated for the off-target editing evaluation are available at BioProject: PRJNA732823.

## Supplemental information

Document 1. Figures S1–S5 and Tables S2 and S5

Table S1. Oligos used in the study

Table S3. Results of 3′ RACE experiment

Table S4. Table of predicted off-target sites by nSpABEmax

Document 2. Article plus supplemental information
