# Supplementary material for: Adenine base editing of the DUX4 polyadenylation signal for targeted genetic therapy in facioscapulohumeral muscular dystrophy
Source: Mol Ther Nucleic Acids. 2021 Jun 1;25:342–54. doi: 10.1016/j.omtn.2021.05.020 (PMC8399085; doi:10.1016/j.omtn.2021.05.020)
Supplement: Document 1. Figures S1–S5 and Tables S2 and S5 [file mmc1.pdf]

OMTN, Volume *25*

Supplemental information

Adenine base editing of the *DUX4* polyadenylation signal for targeted genetic therapy in facioscapulohumeral muscular dystrophy

Darina Šikrová, Vlad A. Cadar, Yavuz Ariyurek, Jeroen F.J. Laros, Judit Balog, and Silvère M. van der Maarel

Supplemental Materials

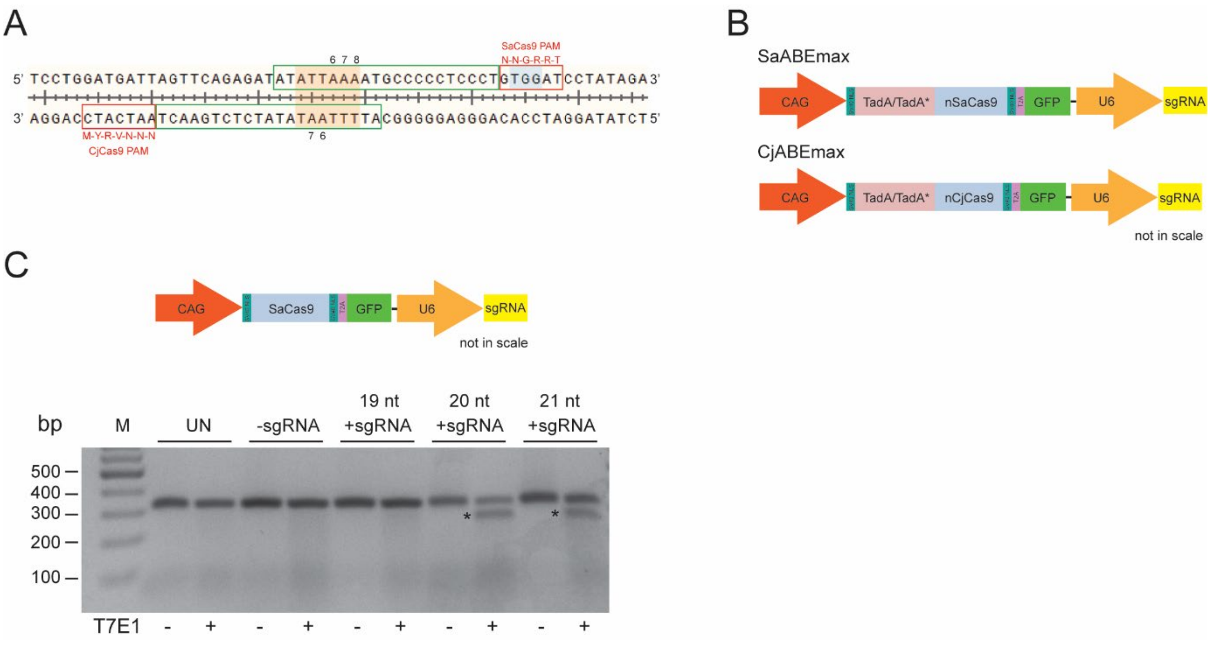

Suppl. Figure 1. *DUX4* PAS is not editable either by nSaABEmax or by nCjABEmax. A) DNA sequence surrounding of *DUX4* PAS (highlighted in the orange box). Cognate PAM sites for CjCas9 and SaCas9 are outlined in red rectangles, while sgRNA protospacer regions are outlined in green rectangles. The PAM site for SpCas9 is highlighted in the blue box. Adenines within the *DUX4* PAS amenable for base editing are numbered from the beginning of the sgRNA protospacer. **B)** Schematic maps of modified all-in-one vectors coding for nSaABEmax (top) and nCjABEmax (bottom). **C)** Schematic map of the pX601 vector for simultaneous sgRNA and SaCas9 nuclease expression (top). Result of the T7E1 assay performed on HAP1 cells which were transfected with a pX601 vector expressing the SaCas9 nuclease and sgRNAs of different length (19 nt-, 20 nt- or 21 nt-long) targeting the *DUX4* PAS (bottom). Untransfected cells (UN) or cells transfected with no sgRNA containing vector (-sgRNA) served as negative control. Asterisks mark the T7E1 cleavage products.

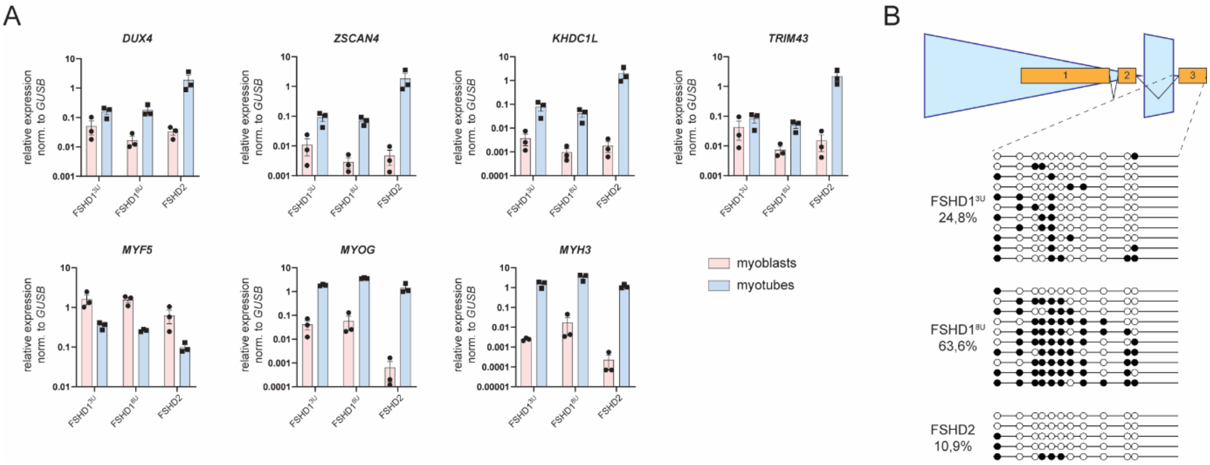

Suppl. Figure 2. Expression and methylation profiles of FSHD immortalized myoblasts used for editing. A) mRNA expression levels of *DUX4* and four DUX4 target genes in 3 model FSHD cell lines used for base editing experiments at myoblast and myotubes stage. Expression of myogenic markers (*MYOG* and *MYH3*) is provided to show successful myogenic differentiation. *GUSB* was used as a housekeeping gene. Bars represent mean ±SEM. Cells were grown three independent times and analysed for their gene expression. **B)** CpG methylation level of the FasPAS region encompassing exon 3 of *DUX4* in the three parental FSHD immortalized myoblast lines used for base editing. Individual rows represent a single molecule, empty circles denote unmethylated cytosines in a CpG context, while full circles denote methylated cytosines in a CpG context. Average methylation of the region (in %) is provided below the name of each sample. Note, that in case of FSHD1^{8U}, both alleles (contracted and non-contracted allele) are amplified in bisulfite PCR.

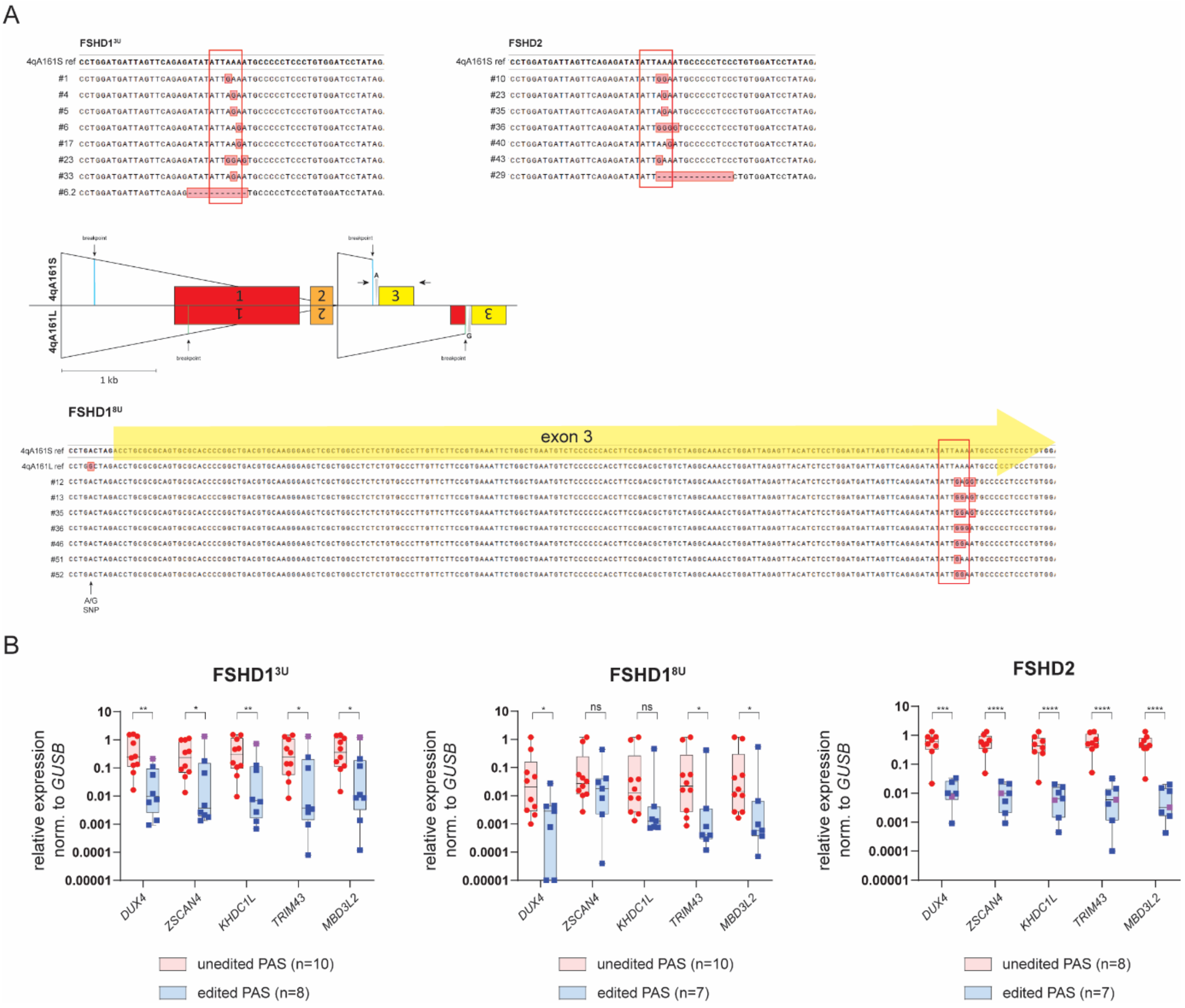

Suppl. Figure 3. Genotypes of successfully edited clones and their expression data in proliferating myoblasts. A) Genotypes of successfully *DUX4* PAS edited clones obtained from three independent FSHD lines (top left: FSHD1^{3U}, top right: FSHD2 and bottom: FSHD1^{8U}) aligned to the WT reference sequence. The *DUX4* PAS sequence is highlighted in a red rectangle and red colored bases denote mismatches. Mirror schematic of the 4qA161S and 4qA161L D4Z4 haplotype termini is provided to show the genotyping approach for the FSHD1^{8u} cell line. Red box represents exon 1, orange box represents exon 2 and yellow box represents exon 3 which corresponds to the yellow arrow highlighting the exon 3 sequence in the genotyping tracks. In the 4qA161L haplotype, a small 5' part of exon 1 (red box) precedes exon 3 due to a different breakpoint. A specific forward primer was used to selectively amplify the 4qA161S allele, which was confirmed by the presence of the SNP (A instead of G) in the Sanger sequencing tracks (marked by arrow) for all the genotyped clones. Reference

sequence for both, 4qA161S and 4qA161L allele is provided. B**)** mRNA levels as assessed by RT-qPCR of *DUX4* and four DUX4 target genes (*MBD3L2*, *ZSCAN4*, *TRIM43* and *KHDC1L*) in PAS unedited vs edited clones derived from two FSHD1 and one FSHD2 cell lines during proliferation. Statistical significance was calculated with unpaired two-tailed t-test (ns: non-significant, *: <0.05, **: <0.01, ***: <0.001, ****: <0.0001) on log2 transformed expression values to correct for skewed distribution. Expression values normalized to *GUSB* as house-keeping gene are plotted. Line represent mean and whiskers represent min and max value. Individual data points represent individual clones, two violet clones carry a deletion over *DUX4* PAS.

A

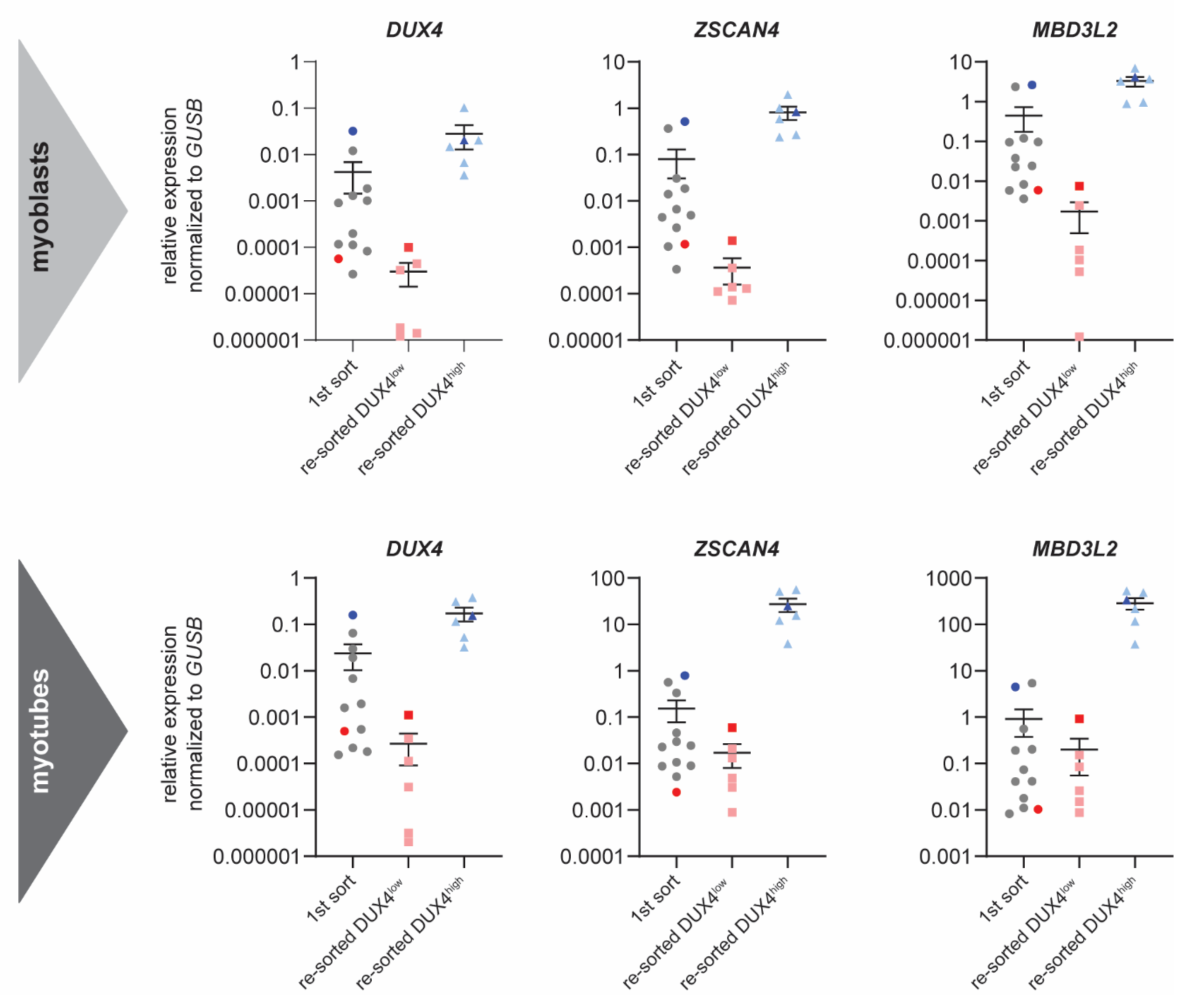

Suppl. Figure 4. DUX4 expression signature is clonally stable. A) mRNA levels as assessed by RT-qPCR of *DUX4* and its two target genes (*ZSCAN4* and *MBD3L2*) were measured in clonal lines established from FSHD1^{8U} immortalized myoblasts and 5 new daughter clones derived from a parental clone with either high *DUX4* expression (dark blue colour) or low *DUX4* expression (dark red colour). Daughter clones are marked by light blue or light red colour. Expression data for both myoblasts (top) and myotubes (bottom) are provided.

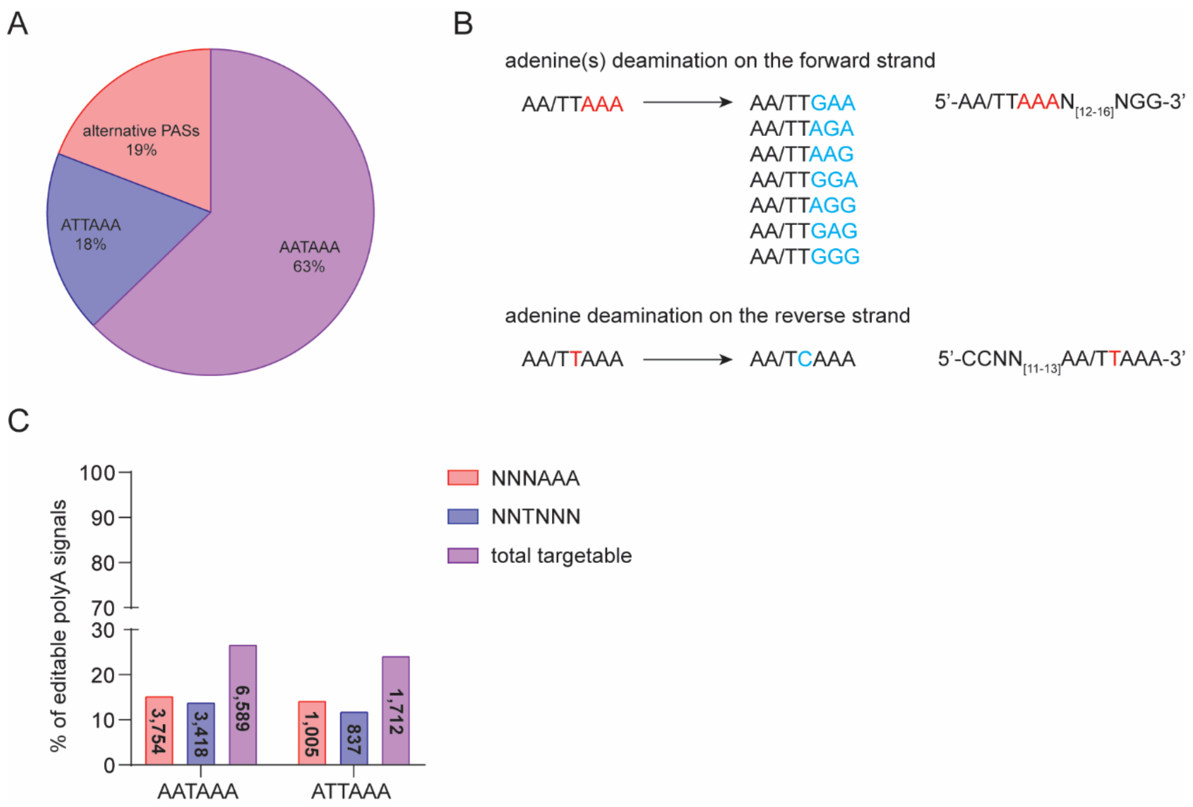

Suppl. Figure 5. A) Identification of polyadenylation signals targetable by nSpABEmax in the human genome. A) Genome-wide prevalence of most common polyadenylation signal hexamers based on all annotated polyadenylation signals in Gencode. **B)** Representation of two different approaches of targeting polyadenylation signals by nSpABEmax either on the coding or non-coding strand and their possible outcomes. Targeted positions are in red and expected modified bases are in blue. **C)** Percentage of annotated polyadenylation signals in the GRCh38 human genome with the most prevalent motifs (AATAAA and ATTAAA) whose adenines are targetable by nSpABEmax either on the coding (red) or non-coding (blue) strand. The number within each bar represents the actual number of targetable polyadenylation signals.

Supplemental Table 1. Oligos used in the study. Table is available online as a separate excel sheet.

Supplemental Table 2. Detailed information about cell lines used in the study.

Cell Line (ID)	Cell Type	Clinical Status and *SMCHD1* Mutation Status	Sex	4q allele #1	4q allele #2
HAP1	derived from chronic myelogenous leukemia (KBM-7)	NA	M	25U 4qA161S	NA
2402 (FSHD3U)	immortalized myoblasts	FSHD1	M	3U 4qA161S	16U 4qB163
073 (FSHD8U)	immortalized myoblasts	FSHD1	M	7U 4qA161S	36U 4qA161L
200 (FSHD2)	immortalized myoblasts	FSHD2 (SMCHD1 p.Lys204Glu)	M	11U 4qA161S	39U 4qB168

Supplemental Table 3. Results of 3' RACE experiment. Calculation table is available online as a separate excel sheet.

Supplemental Table 4. Table of predicted off-target sites by nSpABEmax. Table is available online a as separate excel sheet.

Supplemental Table 5. Summary of efficiencies of deriving edited clones in immortalized myoblasts.

	# of post-FACS grown-out clones	# of edited clones	% efficiency (100*edited/all grown-out)
**$FSHD1^{3U}$ poly**	40	8	20
**$FSHD1^{8U}$ poly**	52	7	13,46153846
**FSHD2 poly**	46	7	15,2173913
**$FSHD1^{3U}$ mono**	8	5	62,5
**$FSHD1^{8U}$ mono**	17	3	17,64705882
**total**	163	30	18,40490798
